# Indicator-focussed technical assistance in South Africa’s HIV programme: A stepped-wedge evaluation

**DOI:** 10.4102/sajhivmed.v22i1.1229

**Published:** 2021-06-15

**Authors:** Geoffrey A. Jobson, Jean Railton, Barry Mutasa, Lucy Ranoto, Christine Maluleke, James McIntyre, Helen Struthers, Remco Peters

**Affiliations:** 1 Programme Management, Anova Health Institute, Johannesburg, South Africa; 2 School of Public Health and Family Medicine, Faculty of Health Sciences, University of Cape Town, Cape Town, South Africa; 3 Division of Infectious Diseases and HIV Medicine, Department of Medicine, University of Cape Town, Cape Town, South Africa; 4 Department of Medical Microbiology, School of Medicine, University of Pretoria, Pretoria, South Africa

**Keywords:** HIV, technical assistance, routine data, stepped wedge, retention in care, TB

## Abstract

**Background:**

There is a lack of research on technical assistance (TA) interventions in low- and middle-income countries. Variation in local contexts requires tailor-made approaches to TA that are structured and replicable across intervention sites whilst retaining the flexibility to adapt to local contexts. We developed a systematic process of TA using multidisciplinary roving teams to provide support across the various elements comprising local HIV services.

**Objectives:**

To examine the effectiveness of targeting specific HIV and TB programme indicators for improvement using roving teams.

**Method:**

We conducted a cluster-randomised stepped-wedge evaluation of a TA support package focussing on clinical, managerial and pharmacy services in the Mopani district of the Limpopo province, South Africa (SA). Three roving teams delivered the intervention. Seventeen primary and community healthcare centres that had 400–600 patients on antiretroviral therapy (ART) were selected for inclusion. The TA package was implemented for six consecutive months across facilities until all had received the same level of support. Data were collected from the relevant health management information systems for 11 routine indicators.

**Results:**

The mean proportion of PLWH screened for tuberculosis (TB) at ART initiation increased from 85.2% to 87.2% (*P* = 0.65). Rates of retention in care improved, with the mean proportion of patients retained in care at three months post-ART initiation increasing from 79.9% to 87.4% (*P* < 0.001) and from 70.3% to 77.7% (*P* < 0.01) after six months. Finally, the mean proportion of patients with TB who completed their treatment increased from 80.6% to 82.1% (*P* = 0.75).

**Conclusion:**

Tailored TA interventions in SA using a standardised structure and process led to a significant improvement in retention-in-care rates and to non-significant improvements in the proportion of PLWH screened for TB and of those who completed their treatment.

## Introduction

Technical assistance (TA) is an important component of many interventions in low- and middle-income countries (LMICs) that promote health systems strengthening (HSS). In South Africa (SA), TA has been implemented in the context of partnerships with international non-governmental organisations (INGOs) and global health initiatives (GHIs) such as the United Sates (US) President’s Emergency Plan for AIDS Relief (PEPFAR) and the Global Fund to Fight AIDS, Tuberculosis, and Malaria (the Global Fund).

The relative importance of TA in HIV response in SA has been directly affected by shifts in PEPFAR policy.^1^ The US President’s Emergency Plan for AIDS Relief’s initial support focussed on building treatment programmes and scaling up access to antiretroviral therapy (ART) through direct service provision.^2^ In 2009 its focus changed to implementing TA that supported the eventual transition of ownership of PEPFAR’s initiatives to the Government of SA. In 2016 PEPFAR’s strategy shifted further to focus on front-line services in districts with a high HIV prevalence, whilst maintaining targeted TA to support specific aspects of the HIV programme.^1^

The implementation of TA is frequently understood in terms of two key concepts: market ‘pull-TA’ and ‘push-TA’.^3,4,5^ ‘Pull-TA’ is based on reactive responses to requests for support, whereas ‘push-TA’ proactively integrates technical expertise, research findings or technology into the programme-level practice.^4^

Implementing TA usually involves a combination of both.^4^ In considering the implementation of TA, Le et al.^6^ suggested a model based on a continuum between ‘content-driven’ and ‘relationship-based’ TA. Content-driven TA emphasises information transfer, capacity building and the increase of the recipients’ knowledge.^6^ Its process tends to be standardised, using predefined strategies to achieve specific goals or targets.^6^

Relationship-based TA tends to be utilised when goals include complex, behavioural and systems change, and shared learning.^6^ Its process is more dynamic and adaptable and is jointly developed and implemented by TA implementers and recipients.^6^

Kredo et al.^7^ noted that in addressing gaps in translating evidence into practice in healthcare settings, tailored, multifaceted approaches that address specific issues seem to be most effective in facilitating change. The healthcare system in SA varies widely in terms of staff capacity, availability of resources and local social, cultural and geographical contexts.^8^ In SA, challenges include financial, budgetary, resource constraints, unreliable drug supplies, poor quality of the physical infrastructure, challenges in governance and leadership, human resource constraints and a lack of capacity amongst front-line staff members.^7,8^ The variation in the severity of specific local challenges suggest that effective TA must be tailored to local health-related constraints and contexts. Access to and the quality of healthcare in LMICs must be addressed if improvements in patient outcomes are to be realised.^9,10^

Tailoring TA therefore requires that specific barriers affecting the provision of health services be identified and that these can be influenced by external inputs from TA providers. These barriers relate to staff capacity, the management of facility-based processes such as patient flow, data management and stock control and in some cases the physical infrastructure of health facilities. Baker et al.^11^ noted that evidence for tailored interventions shows only a limited improvement in professional practice in health systems and indicated a need for research that focusses on more systematic approaches to the tailoring of interventions.

This article reports the results of a study focussing on the implementation of a targeted package of TA for HIV and tuberculosis (TB) treatment and care in the Limpopo province of SA.

## Context and description of the technical assistance intervention package

The Mopani district in the Limpopo province is designated as one of PEPFAR’s 27 priority districts. Priority districts account for ±80% of South Africans living with HIV and are critical to achieving epidemic control in the country.^12^ The population of Mopani was estimated to be ±1.2 million in 2020.^13^ The HIV prevalence amongst adults in the Limpopo province as a whole was estimated at 11.4% in 2017.^14^ There were 106 primary healthcare clinics, 6 district hospitals and 1 regional hospital in the Mopani district at the time of the implementation of the package.

The Anova Health Institute has supported the SA Department of Health (DOH) in the Mopani district since 2004.

Initial funding focussed on direct service delivery via the provision of HIV care and treatment at selected primary care clinics and hospitals. Following PEPFAR’s 2009/2010 re-direction of focus, Anova provided TA primarily to facility managers, individual professional nurses, counsellors and data staff at primary care facilities. The aim was to provide comprehensive HIV and TB care as per the DOH’s strategic plans and policies. At the time these included ART initiation in people living with HIV (PLWH) with CD4 counts of ≤ 500 cells/*µ*L, the immediate initiation of ART in those with active TB, and in pregnant and breastfeeding women living with HIV and those co-infected with the hepatitis B virus (HBV) and HIV.^15^

Each team included a professional nurse, data quality mentors, a pharmacist assistant and a lay counsellor.

Teams were supervised by a medical doctor. An additional technical team provided expert support to the roving teams and worked closely with provincial and district management.

Initially, support was provided on an unstructured basis, with teams responding to needs highlighted by facility staff members during visits. Although this ‘pull-phase’ of support was useful to health facilities,^16^ a lack of clear criteria defining the TA intervention made guiding the roving teams and evaluating their impact challenging. This led to the implementation of facility-level data audits by Anova to identify problematic areas and gaps in the provision of care. Based on this analysis, a package of interventions that focussed on improving specific problem indicators was developed. By tailoring the intervention to the indicators, the roving teams were able to shift to a ‘push-based’ model of TA, providing systematic and measurable programme support to the DOH (see Table 1).

**TABLE 1:** Roving team technical support.

Support process	
Planning	Baseline assessment: Facility audit of HIV and TB files, Tier.net and TB registers and an assessment of the process flow of patients through the clinic. The auditing process involves checking the level of completeness, consistency and accuracy of data from one source to another. Identification of gaps. Meet with the facility manager to present audit results. Develop a quality improvement plan in collaboration with the facility manager.
Implementation	Provide clinical in-service training and mentoring based on the gaps identified in: HIV counselling and testing Linkage to care Clinical care Data quality Drug supply management ART dosing and dispensing
Monitoring and support	Assessment of NIMART mentorship logbooks with clinical mentorship of complicated cases Support to facility staff to conduct monthly facilitation of meetings with CHW/Tracer teams to distribute defaulter lists and get feedback on their tracing efforts.

TB, tuberculosis; ART, antiretroviral therapy; NIMART, Nurse Initiated and Managed Antiretroviral Therapy; CHW, community health workers.

## Methods

The objective of this research study was to examine the effectiveness of targeting specific HIV and TB programme indicators for improvement using roving teams. The hypothesis was that the use of routine data to focus TA on specific indicators would facilitate the identification of areas of practice requiring support and would lead to improvements in health service delivery.

### Study design

This was a randomised, stepped-wedge evaluation in which each healthcare facility was considered a cluster.

A stepped-wedge design is a variant of cluster-randomised trial designs in which clusters included in the sample are randomly allocated a time when they are given the intervention.^17^ Clusters cross over from control to intervention at regular intervals until all clusters have received the intervention (see Table 2). A stepped-wedge design was chosen because it enabled the use of a randomised controlled approach alongside the implementation process. Further, it would have been unethical to exclude facilities from receiving support as would be the case in a standard randomised controlled trial (RCT). In terms of logistics, it would have been impossible to deliver the intervention simultaneously to all facilities.

**TABLE 2:** Sequence of support implementation.

Facility	Month (2015)							
	Jan	Feb	Mar	April	May	June	July	Aug
Dan Village	SS	SS	IS	IS	MS	MS	MS	MS
Sekgopo	SS	SS	IS	IS	MS	MS	MS	MS
Namakgale B	SS	SS	IS	IS	MS	MS	MS	MS
Dr Hugo	SS	SS	IS	IS	MS	MS	MS	MS
Seapole	SS	SS	IS	IS	MS	MS	MS	MS
Mariveni	SS	SS	IS	IS	MS	MS	MS	MS
Maphalle	SS	SS	SS	SS	IS	IS	MS	MS
Makhushane	SS	SS	SS	SS	IS	IS	MS	MS
Shiluvana	SS	SS	SS	SS	IS	IS	MS	MS
Bellevue	SS	SS	SS	SS	IS	IS	MS	MS
Letsitele	SS	SS	SS	SS	IS	IS	MS	MS
Matswi	SS	SS	SS	SS	IS	IS	MS	MS
Humulani	SS	SS	SS	SS	SS	SS	IS	IS
Morutji	SS	SS	SS	SS	SS	SS	IS	IS
Duiwelskloof	SS	SS	SS	SS	SS	SS	IS	IS
Lephepane	SS	SS	SS	SS	SS	SS	IS	IS
Meedingen	SS	SS	SS	SS	SS	SS	IS	IS

SS, standard support; IS, intensive support; MS, maintenance support.

Support was implemented in stages. Each facility received two months of intensive support, followed by lower-intensity maintenance support over the following 4 months. Results of the TA intervention were monitored using routine programme data from the relevant health management information systems (HMIS) that included the Three Integrated Electronic Registers system (TIER.net) and the District Health Information System (DHIS); data were drawn from TIER.net and facility-level paper registers. The DHIS assisted with the verification of statistics collected from the registers and from the TIER.net. The latter is a three-tiered monitoring system using paper registers (tier 1), an offline electronic register (tier 2) and networked electronic medical records (tier 3).^18^ The specific ‘tier’ used depended on the context and resources available at the time of implementation. Allowing a phased approach to the implementation of electronic medical records across the health system as a whole.^19^ A dedicated data capturer logged specific indicators from the clinic file into the TIER.net after every patient visit. The DHIS is an aggregated reporting system and indicators are captured at a facility level. Some of the data elements and indicators that are captured or exported in DHIS are generated from the TIER.net and some from longitudinal or tick registers.

The randomisation step refers to a point at the time of implementation of the support package at each facility. The clusters are of relatively similar size to avoid bias and to reduce the need for correction in analysis. Data collection occurred from January to October 2015.

### Sampling

All 64 primary healthcare (PHC) and community healthcare (CHC) facilities in Greater Tzaneen (33), Greater Letaba (21) and Ba-Phalaborwa (10) subdistricts of the Mopani district were listed. These subdistricts were chosen primarily for logistical reasons as they were more easily accessible to the doctors who were supporting the roving teams. The main difference between the PHC and CHC facilities is the level of service offered. Primary healthcare centres provide only primary health services, whilst CHCs should ideally have a full-time doctor and offer 24-h maternity and emergency services.^20^

To include clusters of similar size, we selected facilities with 400–600 patients on ART in March 2014. Randomisation of facilities was performed using random number lists and resulted in the stepped-wedge sequence of support implementation shown in Table 2. A total of 17 facilities were included in the study sample.

### Data analysis

This study used routinely available data. Data were collected for 11 indicators that were used for the operational implementation of the TA programme (see Table 3).

**TABLE 3:** Outcome measures.

Programme	Indicator	Indicator definition
**Domain 1: HIV testing and ART initiation**		
HIV	Proportion of eligible HIV-infected individuals started on ART	Number of newly diagnosed eligible HIV-infected individuals starting ART during the reporting period as a percentage of the total number of newly diagnosed patients during the reporting period
TB	Proportion of adults screened for TB at the start of ART	Proportion of patients not on tuberculosis (TB) treatment who were screened for TB at the start of ART
TB	Proportion of TB patients tested for HIV	Number of registered TB patients tested for HIV as a proportion of all registered TB patients
TB	Proportion of TB and HIV co-infected patients initiated on ART	Number of TB patients who were HIV-positive and who were not on ART and started on ART as a proportion of all TB patients who were HIV-positive and were not on ART at the start of TB treatment
**Domain 2: ART monitoring and programme performance**		
HIV	Proportion of adults retained in ART care at 3 and 6 months	Number of adult patients who remained on ART and had started ART 3 or 6 months prior to the reporting period as a percentage of the total adults who started treatment 3 or 6 months prior to the reporting period
HIV	Proportion of adults on ART who were lost to follow-up at 3 and 6 months	Number of adult patients who were declared lost to follow-up by the end of 3 or 6 months after starting treatment as a percentage to the total adults who started treatment 3 or 6 months prior to the reporting period
HIV	Proportion of adults with viral load completion at 12 and 24 months	Proportion of adults on ART in the 12- or 24-month cohorts who had a viral load test performed in the last 12 months
HIV	Proportion of adults with viral load suppression at 12 and 24 months	Number of adults on ART in the 12- or 24-month cohorts who had a viral load of <400 cp/mL as a proportion of adults on ART with viral load completion
**Domain 3: TB programme performance**		
TB	TB (new pulmonary) patients initiated on TB treatment	New TB patients started on drug susceptible tuberculosis (DS-TB) treatment as a proportion of all TB patients started on DS-TB treatment
TB	TB (new pulmonary) treatment success rate	Number of registered TB patients on TB treatment and were cured as a proportion of all registered TB patients
TB	TB (new pulmonary) defaulter rate	Number of registered TB patients on TB treatment who defaulted as a proportion of all registered TB patients

*Source:* South African Department of Health. Ideal community health centre: Definitions, components and checklists. Pretoria: South African Department of Health. 2020.

ART, antiretroviral therapy; TB, tuberculosis.

Data were analysed using STATA version 12, with a significance threshold of 0.05 for statistical tests.^21^ Although data for all indicators were collected, only four of these indicators included a sufficiently large number of patients to allow for the measurement of effects in our analyses. Also, whilst the indicator for the number of eligible PLWH initiated on ART included a sufficient number of patients, the registers used to document these patients changed during the course of programme implementation, which complicated the attempts to verify the data.

Subsequently we decided to exclude this indicator from our analysis. The indicators that were used for effect analysis included proportion of eligible HIV-infected adults screened for TB at the start of ART, proportion of adults on ART retained in care at three months, proportion of adults on ART retained in care at six months and TB treatment success rate.

Analyses used total patient numbers across facilities and examined changes in proportions for each indicator for pre- and post-TA implementation support. Descriptive data included the total number of patients eligible for inclusion in each indicator (pre-support), the total number of patients successfully completing each indicator, per cent completion and mean completion. Statistical significance of changes in the proportion of patients successfully completing each indicator was measured using two sample *t*-tests with unequal variances.

### Ethical considerations

Ethical approval for the study was obtained from the University of the Witwatersrand Human Research Ethics Committee (MP 140949) and the Limpopo Provincial Health Research Committee.

## Results

The TA package was successfully implemented, with a total of 515 TA visits conducted over the study period.

Clinical services were the most frequent reasons for TA across all three phases of implementation, comprising 256 (49.7%) of total visits, with most of these focussing on clinical mentoring (83; 32.4%) or in-service training (72; 28%) (see Table 4). These aspects of support focussed on facility-level factors that affected particular indicators and hence varied between facilities. For example, a factor contributing to lower-than-expected ART initiation rates may have been the monitoring of CD4 counts (prior to the implementation of immediate initiation of ART following HIV-positive test results). In such a case, mentoring would focus on establishing procedures for regular monitoring of CD4 counts in pre-ART patients.

**TABLE 4:** Technical assistance support activities and number of visits.

Type of support	Activity	Total visits per support phase			Grand total
		Phase 1	Phase 2	Phase 3	
**Clinical services support**	Audits/assessment	22	21	9	52
	Clinical mentoring visit	36	31	16	83
	HCT mentoring visit	31	14	4	49
	In-service training	26	29	17	72
**Total clinical services support visits**		115	95	46	256
**Managerial support**	District/subdistrict interventions	5	9	6	20
	Feedback meetings with operational managers	9	11	3	23
	Ward-based outreach team meeting	12	6	1	19
**Managerial support total**		26	26	10	62
**Non-clinical services support**	M&E direct service delivery	23	24	10	57
	M&E mentoring visits	22	24	10	56
	Pharmacy mentoring visits	40	30	14	84
**Non-clinical services support total**		85	78	34	197
**Grand total**		226	199	90	515

HCT, HIV counceling and testing; M&E, monitoring and evaluation.

Technical assistance for monitoring and evaluation (M&E) and pharmacies made up 197 (38.3%) of the total visits, whilst 62 (12%) visits focussed on managerial support (Table 4). These types of support also varied in focus depending on the specific challenges identified at facilities. For example, M&E support may include training on data verification and file audits for data-entry clerks, whilst managerial support could include developing task-sharing processes for the use of TB-screening tools for lay counsellors and enrolled nurses.

Improvements were observed for all indicators included in the study (Table 5). The mean proportion of HIV-positive adults screened for TB at ART initiation increased from 85.2% (s.d. = 0.036) to 87.2% (s.d. = 0.026), although this change was not significant: *t*(115.8) = –0.45 (*p* = 0.65). Rates of retention in care improved at both 3 and 6 months post-ART initiation. The mean proportion of patients retained in care at 3 months post-ART initiation increased from 79.9% (s.d. = 0.014) to 87.4% (s.d. = 0.011), *t*(118) = –4.28 (*p* < 0.001), and from 70.3% (s.d. = 0.018) to 77.7% (s.d. = 0.016), *t*(126) = –3.12 (*p* < 0.01), after 6 months. Finally, the mean proportion of patients with TB who completed their treatment increased from 80.6% (s.d. = 0.03) to 82.1% (s.d. = 0.033), *t*(135) = –0.32 (*p* = 0.78).

**TABLE 5:** Total and percentage change in indicators pre- and post-support.

Indicator	Without TA support				With TA support			
	Patients eligible	Patients completed	Total percentage	Mean percentage	Patients eligible	Patients completed	Total percentage	Mean percentage
Proportion of HIV-infected adults screened for TB at ART initiation	1337	1104	82.6	85.2	897	788	87.8	87.2^ns^
Proportion of adults on ART retained in care at 3 months	1214	967	79.7	79.9	1175	1027	87.4	88***
Proportion of adults on ART retained in care at 6 months	1055	739	70	70.3	1123	873	77.7	77.8**
TB treatment success rate	262	213	81.3	80.6	246	212	86.2	82.1^ns^

TA, technical assistance; TB, tuberculosis; ART, antiretroviral therapy; ns, not significant.

* , *p* < 0.05;

** , *p* < 0.01;

*** , *p* < 0.001.

## Discussion

Technical assistance in health systems with varying levels of competence and diverse, localised challenges needs to be flexible and adaptable to effectively respond to the specific contexts in which health services operate. As Kredo et al.^7^ noted with regard to the implementation of the Clinical Practice Guidelines (CPG) in the South African context, ‘… for effective CPG implementation in health services to occur, considerations of the unique settings in each province, including culture, geography and social needs, must be undertaken’.

The results of this study provide an example of one way to address the specific local factors affecting health service delivery across the varying contexts of the South African health system. By using a systematic approach to identify localised health system challenges and structuring TA interventions around specific, measurable outcomes directly related to these challenges, the implementing agency was able to support rapid improvements in key programme outcomes, although only the indicators related to retention in care showed significant improvement. This is likely a reflection of the relative complexity and/or resources available in support of the processes underlying the programme indicators. Rates of retention in care may have improved because of the support of community health workers and data clerks in generating defaulter lists and actively contacting patients at risk of exiting care. In contrast, TB treatment success depends to a large extent on individuals’ specific community-level contexts and is less easily influenced by healthcare workers. Similarly, the increased workload and complexity of including screening for TB during ART initiation may have limited the effectiveness of support for this indicator.

There were two shifts in the TA approach that facilitated the improvement: firstly a move from pull to push TA^4^ and secondly a shift from TA based primarily around relationships between roving teams and healthcare workers^16^ to TA that included a stronger focus on more structured content.^6^

The shift from pull to push TA hinged on the implementing agency’s analysis of programme data and their identification of gaps in the delivery of HIV and TB services. This analysis enabled the agency to design targeted, structured interventions guided by actual gaps in service delivery, rather than on an *ad hoc* basis according to requests from healthcare workers and managers. The shift towards a push-based approach to TA also meant that the implementation of support became more structured and content-focussed, with the development of action plans between facility managers and mentors. This contrasted with the relatively *ad hoc* support that had previously been provided by the roving teams in which they responded to concerns raised by facility staff and health workers as problems arose.

The use of a structured systematic approach to identify and address gaps in health service provision also provides a replicable process that can be adapted to a wide range of contexts. This is important because of the broad scope of ‘TA’ interventions.^4^ In addition, the team-based approach used to provide TA enabled the implementing agency to address multiple problematic areas simultaneously across clinical, pharmaceutical and managerial departments. In the context of donor-supported health programmes in LMICs, this approach may facilitate a relatively rapid improvement in key indicators.

Finally, this study points to the potential importance of the use of routine data in programme evaluation processes and to the usefulness of incorporating routine indicators into study designs.^22^ However, for this data to be useful requires HMI systems to be functional, accurate and up-to-date, and it further highlights the importance of HMI systems as one of the World Health Organization’s (WHO) health system building blocks.^23^

### Limitations

The primary limitation of this study is the lack of usable data for the analysis from the HMIS for seven of the 11 indicators measured because of the small numbers of patients accessing the services related to these indicators. Data for all indicators were used to guide TA implementation. This points to the importance of data management and the maintenance of HMIS as critical resources for programme evaluation in rural South African contexts.

## Conclusion

This study demonstrates the potential effectiveness of linking TA interventions in SA to specific, measurable outcomes and highlights the value of developing a structured intervention process that can be adapted to local contexts and challenges. The ability to tailor TA to individual facility needs is particularly important, and further implementation-based research should be conducted to improve the understanding of how to tackle the wide range of context-specific challenges facing health systems in LMICs.
